# O2C-Based Evaluation of Microperfusion During Dialysis Fistula Creation

**DOI:** 10.3390/jcm14196849

**Published:** 2025-09-27

**Authors:** Lillian Schmoll, Andreas L. H. Gerken, Caroline Schröder, Christel Weiß, Christoph Reißfelder, Johannes Eberhard, Niklas Ayasse, Zoi Bougioukou, Sebastian Zach, Kay Schwenke

**Affiliations:** 1 Department of Surgery, University Medical Center Mannheim, Medical Faculty Mannheim, Heidelberg University, Theodor-Kutzer-Ufer 1-3, 68167 Mannheim, Germany; andreas.gerken@umm.de (A.L.H.G.); christoph.reissfelder@umm.de (C.R.); johannes.eberhard@umm.de (J.E.); sebastian.zach@umm.de (S.Z.); kay.schwenke@umm.de (K.S.); 2 Department of Medical Statistics and Biomathematics, University Medical Center Mannheim, Medical Faculty Mannheim, Heidelberg University, Theodor-Kutzer-Ufer 1-3, 68167 Mannheim, Germany; christel.weiss@medma.uni-heidelberg.de; 3 Department of Internal Medicine, Nephrology, Hypertensiology, Transplantation Medicine, Endocrinology, Diabetology, Lipidology, Rheumatology, Pulmonology, Medical Center Mannheim, Medical Faculty Mannheim, Heidelberg University, Theodor-Kutzer-Ufer 1-3, 68167 Mannheim, Germany; niklas.ayasse@umm.de (N.A.); zoi.bougioukou@umm.de (Z.B.)

**Keywords:** O2C (oxygen to see), micro- and macro-perfusion, regional anaesthesia, Dialysis Access Steal Syndrome (DASS), arteriovenous fistula (AVF)

## Abstract

**Background:** Regional anesthesia during arteriovenous fistula (AVF) creation increases arterial and venous diameters and intraoperative flow through sympathetic blockade and vasodilation. These changes are associated with improved short- and medium-term AVF patency. However, their impact on long-term outcomes remains unclear. While current assessments focus on macrocirculatory parameters, no objective method exists to evaluate microcirculatory changes perioperatively. The use of non-invasive optical devices such as the O2C remains investigational in this context. **Methods:** This single-center prospective observational study enrolled 31 patients aged ≥18 years undergoing AVF surgery. Microcirculatory parameters—including tissue oxygen saturation (SO_2_), flow, velocity, and relative hemoglobin concentration (rHb)—were recorded using the non-invasive O2C spectrophotometer (LEA Medizintechnik, Germany). Measurements were performed at the thenar and hypothenar regions at five defined time points, before and after surgery. Where applicable, O2C parameters were compared with shunt flow volumes measured by duplex ultrasonography. **Results:** Plexus anesthesia led to a significant increase in SO_2_, flow, and velocity (*p* < 0.0001). After AVF creation, these parameters declined compared to values under functional regional anesthesia. Correlation between O2C values and ultrasound-measured shunt flow on the first postoperative day was weak (r = 0.40 for flow at M1). **Conclusions:** This is the first study to objectively demonstrate the effects of plexus anesthesia on microcirculation during AVF surgery. O2C may serve as a valuable non-invasive tool to assess perioperative microcirculatory changes.

## 1. Introduction

Arteriovenous fistula (AVF) creation remains the preferred method of vascular access for patients requiring long-term haemodialysis due to its superior patency rates, lower infection risk, and cost-effectiveness compared to other modalities such as central venous catheters or synthetic grafts. However, AVF creation is not without challenges. Early failure, delayed maturation, and complications such as Dialysis Access-associated Steal Syndrome (DASS)—a condition resulting from the diversion of arterial blood away from the distal limb—continue to hinder optimal outcomes and contribute to patient morbidity. These complications are particularly prevalent in patients with borderline vessel calibers or significant comorbidities, such as diabetes or peripheral arterial disease.

In recent years, regional anaesthesia—most notably brachial plexus block—has gained prominence as a strategy to improve surgical and vascular outcomes in AVF surgery. Randomized controlled trials and meta-analyses have demonstrated that regional anaesthesia significantly increases both venous and arterial diameters intraoperatively, as well as the overall intraoperative blood flow. These effects are attributed to sympathetic blockade and the resultant vasodilation, which may be particularly advantageous in patients with compromised vascular reactivity. By improving the hemodynamic profile during fistula creation, regional anaesthesia has been associated with higher rates of short- and mid-term AVF maturation and reduced early failure compared to local anaesthesia—especially in high-risk patient groups [1,2,3,4,5,6,7].

The importance of these findings has been acknowledged in current guidelines. The National Kidney Foundation Kidney Disease Outcomes Quality Initiative (KDOQI) endorses the use of regional anaesthesia as a means of enhancing vascular calibre, thereby facilitating more distal AVF creation. However, the impact of regional anaesthesia on long-term fistula maturation and patency remains less well-defined [8]. Most studies have focused on macrocirculatory endpoints—such as vein diameter, intraoperative flow, or patency—while changes in the microcirculation remain poorly understood.

Despite the well-documented macrocirculatory effects of regional anaesthesia, a major gap persists in the objective, real-time assessment of microcirculatory dynamics during and after AVF creation. The diagnosis and management of DASS are currently based on clinical signs and non-invasive arterial testing (e.g., digital pressures or Doppler studies), but these methods do not directly evaluate tissue-level perfusion. This limits early detection of critical ischemia and may delay intervention [8,9,10,11].

Emerging optical methods, such as the O2C (oxygen to see) device, offer promising avenues for non-invasive microcirculatory monitoring. By combining laser Doppler flowmetry and tissue spectrophotometry, O2C provides real-time quantification of key microcirculatory parameters, including oxygen saturation (SO_2_), relative hemoglobin concentration (rHb), blood flow, and flow velocity. These measurements offer a unique window into tissue-level perfusion and oxygen delivery, potentially bridging the gap between clinical observation and microvascular physiology.

However, the application of O2C in AVF surgery remains investigational. To date, no published studies have systematically assessed the utility of this technology for intraoperative or postoperative monitoring of AVF-related microperfusion. Its role in detecting early signs of DASS, evaluating the effects of regional anaesthesia, or predicting long-term fistula outcomes has not been established in clinical practice. Furthermore, the interpretation of O2C-derived values in the context of AVF surgery is complicated by the lack of standardized reference ranges or outcome thresholds.

Given these limitations, further research is warranted to determine whether microcirculatory monitoring with devices like O2C can improve perioperative assessment, early complication detection, and ultimately, AVF patency. If proven effective, such tools could refine clinical decision-making, facilitate individualized anaesthesia strategies, and provide an objective physiological correlate for the success of AVF procedures

## 2. Materials and Methods

### 2.1. Study Design

This study was designed as a single-center, prospective observational cohort conducted at a tertiary academic vascular surgery department in Mannheim, Germany. Eligible patients were adults aged 18 years or older scheduled to undergo arteriovenous fistula (AVF) creation. Patients were excluded if either the O2C device or a study investigator was unavailable at the time of surgery. Additional exclusions occurred due to COVID-19-related restrictions or if patients declined participation. Between June 2019 and June 2021, we enrolled a total of 31 patients consecutively. The study aimed to achieve a minimum of 15 patients with complete long-term follow-up data, which informed the recruitment duration and sample size. Recruitment was delayed by COVID-19-related clinical and logistical constraints, affecting patient scheduling and procedural availability.

All participants received standard care at our center. The study was conducted in accordance with the Declaration of Helsinki and was approved by the Ethics Committee of the Medical Faculty Mannheim (approval number: 2019-667N). Although patient enrollment began prior to formal registration, the study was retroactively registered in the German Clinical Trials Register (DRKS00037788, registration date: 10 September 2025. Microcirculatory parameters were measured noninvasively using spectrophotometric techniques with the Oxygen-to-see device, version III (Micro-Lightguide O2C^®^, LEA Medizintechnik, Giessen, Germany). Measurements were taken at two anatomical sites—the thenar (M1) and hypothenar (M2) eminences—at five specific time points: (Time 1) preoperatively, before plexus anesthesia; (Time 2) ten minutes after achieving functional plexus anesthesia; (Time 3) immediately postoperatively; (Time 4) on the first postoperative day (POD); and (Time 5) six weeks after surgery. Because of scheduling variability, Time 5 measurements were performed between 6 and 10 weeks postoperatively. In addition to O2C microcirculatory assessment, macrocirculatory perfusion was evaluated at Time 4 and Time 5 using duplex sonography. To minimize measurement bias, all O2C measurements were conducted by trained personnel using a standardized protocol as described below under controlled environmental conditions. However, selection bias cannot be fully excluded due to limited device and personnel availability during COVID-19 and the lack of randomization. Consistent personnel and protocol adherence mitigated observer bias, but blinding was not feasible in this observational setting.

#### Measurement Protocol

All microcirculatory measurements were performed following a standardized protocol using the same O2C device (Micro-Lightguide O2C^®^, LEA Medizintechnik, Giessen, Germany). Patients were positioned in a supine position with their upper limbs resting comfortably alongside the torso and the palms facing upward. To reduce external influences, patients were instructed to remain silent during the measurement process, and ambient lighting was switched off to prevent direct light exposure to the probes. In selected cases, the probes were additionally shielded to ensure consistency and minimize signal disturbance.

**Figure 1:**
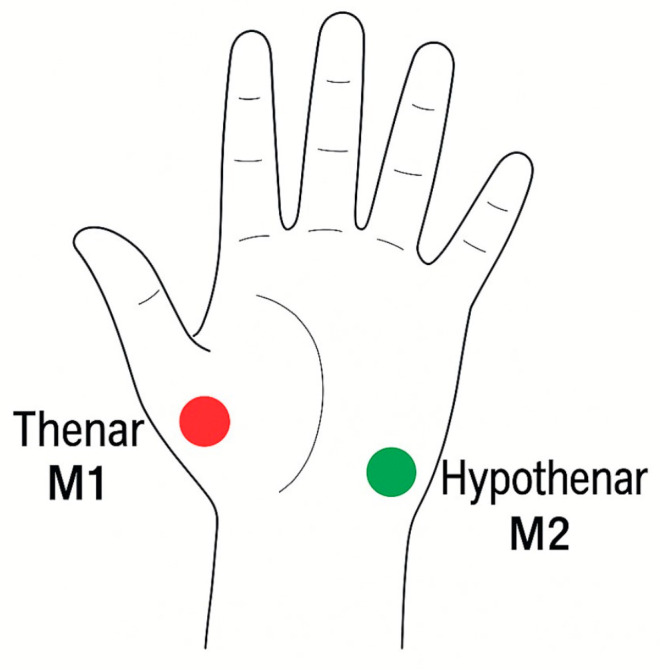
Schematic illustration of the hand showing measurement positions M1 (thenar) and M2 (hypothenar) used on the O2C measurement protocol probe placement.

The LFx29 probe was secured with transparent adhesive tape to the thenar (M1) and hypothenar (M2) regions of the operative hand, as illustrated in Figure 1 below. These anatomical sites were chosen for their reliable representation of palmar microcirculatory flow and for ease of reproducibility across patients.

The measurement protocol builds upon earlier clinical experience with the O2C system for evaluating microvascular dynamics in surgical and intensive care settings [12,13,14]. Their foundational publications demonstrated the feasibility of non-invasive, real-time monitoring of oxygen saturation and perfusion in various clinical contexts, laying the groundwork for subsequent applications of O2C technology in vascular and reconstructive surgery. In particular, their work highlighted the sensitivity of O2C parameters—such as SO_2_, rHb, Flow, and Velocity—to changes in local perfusion pressure and sympathetic tone, reinforcing the relevance of this method for assessing microvascular responses to regional anaesthesia and surgical manipulation.

For the purpose of this study, measurements were taken using a foot pedal. The device performed a 4 s stabilization, after which data were recorded over a 10 s period. The same protocol was followed for both measurement sites. The following microcirculatory parameters were recorded using the O2C device: oxygen saturation (SO_2_, %), relative hemoglobin concentration (rHb, arbitrary units), blood flow (Flow, arbitrary units), and flow velocity (Velocity, arbitrary units).

### 2.2. Principle of the Micro-Lightguide Spectrophotometer (O2C)

In short, the O2C device combines laser Doppler flowmetry and white light spectrometry to measure microcirculatory parameters (Flow, Velocity, SO_2_, rHb). A continuous-wave laser (830 nm, 30 mW) penetrates tissue, and the Doppler shift in the backscattered signal from moving erythrocytes is analysed to determine blood flow velocity and relative flow. White light (450–850 nm) is used to assess oxygen saturation based on differential absorption by oxy- and deoxyhaemoglobin. The relative haemoglobin concentration is derived from the total absorption spectrum.

### 2.3. Statistical Analysis

We performed statistical analyses using SAS software version 9.4. To assess changes associated with regional block anesthesia, we applied the Mixed Procedure. A *p*-value < 0.05 was considered statistically significant. We calculated mean values and standard deviations for all parameters. Subgroup analyses (e.g., diabetic vs. non-diabetic patients) were performed using two-sample *t*-tests. Outliers were identified based on Z-transformation, with values > 2 or <−2 standard deviations from the mean flagged as outliers.

To explore potential predictors of Dialysis Access Steal Syndrome (DASS), we conducted logistic regression analysis using intraoperative and postoperative microcirculatory parameters. Receiver Operating Characteristic (ROC) analysis was used to assess model performance, and area under the curve (AUC) was reported with corresponding 95% confidence intervals (CI).

## 3. Results

### 3.1. Patient Cohort and Baseline Characteristics

**Figure 2:**
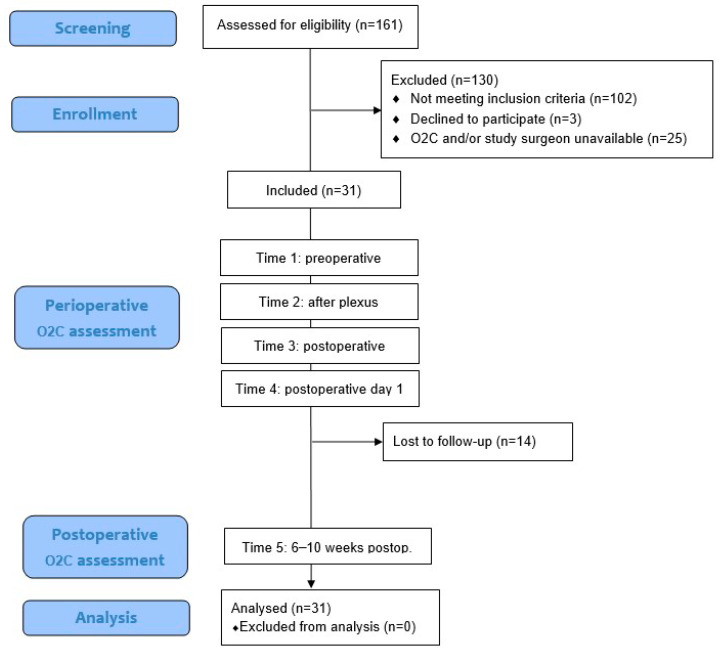
Flow diagram illustrating the stages of patient screening, enrollment, measurement, and follow-up in this prospective observational study. A total of 161 patients undergoing AVF creation were screened. After applying exclusion criteria (e.g., COVID-19-related limitations, non-availability of the study team, patient refusal), 31 patients were enrolled. Longitudinal measurements were conducted perioperatively, and 17 patients completed long-term follow-up.

Between June 2019 and June 2021, a total of 161 patients who underwent creation of a cubital arteriovenous haemodialysis access at our center were screened for eligibility. Of these, 102 patients were excluded because they did not meet the inclusion criteria. An additional 3 patients declined to participate, and in 25 cases, the O2C device and/or study surgeon were not available due to logistical constraints or COVID-19-related limitations. Ultimately, 31 patients were enrolled in this prospective observational study. O2C measurements were obtained at four aforementioned perioperative time points for all patients. A follow-up measurement at 6–10 weeks postoperatively (Time 5) was conducted in 17 patients, while 14 patients were lost to follow-up. Additionally, 30 patients underwent duplex sonographic measurement of shunt flow on POD1. The sample size of 31 was determined to ensure that at least 15 patients would complete the long-term follow-up, which guided the duration of recruitment. All enrolled patients were included in the final analysis. The following flow diagram (Figure 2) visualizes the different stages of patient screening, enrollment, measurement, and follow-up within the study cohort.

A total of 31 patients were recruited for the study, with 51.6% (*n* = 16) being female. An overview of the demographic characteristics is shown in Table 1.

Relevant comorbidities and cardiovascular risk factors were common in the study population. Nearly half of the patients (48.4%) had a history of smoking, either current or former, with significant cumulative exposure (median 45 and 20 pack-years, respectively). Peripheral arterial occlusive disease was present in 12.9%, and 25.8% had a history of coronary artery disease. Almost all patients (96.8%) were hypertensive. The underlying cause of End Stage Renal Disease (ESRD) was identified in 51.6% of patients, with polycystic kidney disease, heminephrectomy, and IgA nephritis being the most frequent causes. Less common causes included undefined vasculopathy, chronic glomerulonephritis, nephritic syndrome, and vancomycin toxicity. Additional details of comorbidities and risk factors are provided in Table 2.

The surgical procedures included both primary (*n* = 23) and secondary (*n* = 8) vascular access creations, performed mainly under regional anesthesia. Various fistula types were utilized, with Gracz fistulas being the most common. The left arm was preferred over the right for access creation. Details regarding the types of access, anesthesia, procedure duration, and postoperative flow measurements are summarized in Table 3 below.

### 3.2. Microcirculatory Changes Induced by Regional Anaesthesia

To evaluate how regional anaesthesia impacts microcirculatory parameters measured by the O2C device, we analysed how time, measurement region, and their interaction influence SO_2_, rHb, Flow, and Velocity. The effect of time shows changes in each parameter before and after administering regional anaesthesia, while the effect of region compares measurements between the thenar (M1) and hypothenar (M2) sites. The interaction term (time × region) assesses whether the response to anaesthesia varies depending on the measurement site.

**Figure 3:**
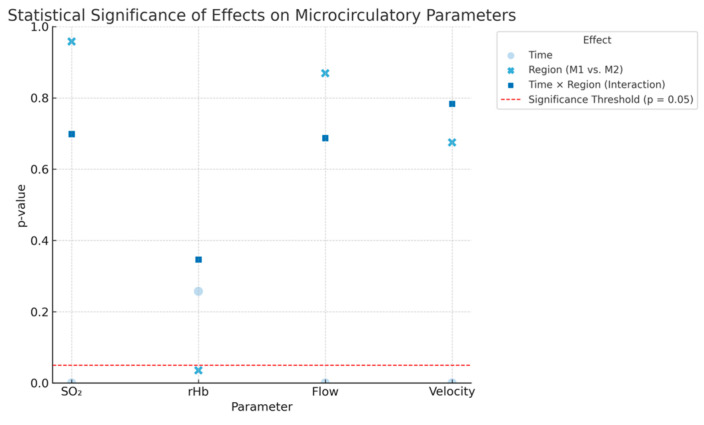
Statistical significance of effects on microcirculatory parameters. *p*-values for the effects of Time, Region (Thenar [M1] vs. Hypothenar [M2]), and their Interaction on four microcirculatory parameters: oxygen saturation (SO_2_), relative hemoglobin (rHb), blood flow, and flow velocity. The horizontal red dashed line indicates the significance threshold (*p* = 0.05). Time had a statistically significant effect on SO_2_, Flow, and Velocity (all *p* < 0.0001), while a weak regional effect was observed for rHb (*p* = 0.0355). No significant interaction effects were detected for any parameter. This figure visualizes the overall statistical model results and highlights the dominance of temporal changes in response to regional anesthesia.

A highly significant effect of time was observed for SO_2_, Flow, and Velocity (all *p* < 0.0001), indicating a consistent increase in these parameters following regional anaesthesia. A weak but statistically significant regional effect was found only for rHb (*p* = 0.0355), while no significant interaction between time and region was observed for any parameter. These findings suggest a general enhancement of microcirculation, particularly in terms of oxygen saturation, blood flow, and flow velocity, following regional anaesthesia, regardless of the measurement site. Detailed results are shown in Figure 3 below.

### 3.3. Paired Comparison of Time Points

#### 3.3.1. Time 1 (Pre-Anaesthesia) vs. Time 3 (Immediately Postoperative)

Regional anaesthesia and surgery resulted in a highly significant increase in all measured parameters—SO_2_, rHb, Flow, and Velocity (*p* < 0.0001 for each). Only rHb showed a significant difference between the thenar and hypothenar regions (*p* = 0.0068). We found no significant interaction between time and region for any parameter.

#### 3.3.2. Time 2 (Post-Regional Block) vs. Time 3 (Immediately Postoperative)

SO_2_ and rHb continued to increase significantly after the surgical intervention (*p* < 0.0001), and Velocity also increased significantly (*p* = 0.0019). Flow showed a trend toward significance (*p* = 0.0898). Once again, we found no significant interaction between time and region.

#### 3.3.3. Time 1 (Pre-Anaesthesia) vs. Time 4 (First Postoperative Day)

rHb increased significantly over time (*p* = 0.0001), and we observed a marginally significant regional difference (*p* = 0.0285). SO_2_, Flow, and Velocity did not change significantly at this time point, and we found no significant interaction effects.

#### 3.3.4. Time 1 (Pre-Anaesthesia) vs. Time 5 (Long-Term Follow-Up)

Only rHb showed a significant long-term increase (*p* = 0.0040). SO_2_, Flow, and Velocity remained stable over time. We found no significant regional differences or time–region interaction effects for any parameter.

### 3.4. Changes in Microcirculation Associated with Diabetes Mellitus

We analysed the impact of diabetes mellitus on microcirculatory parameters using mixed-effects models at both measurement sites (M1: thenar, M2: hypothenar). The analysis included diabetes status (insulin-dependent and non-insulin-dependent) and time points 1, 4, and 5. We observed a significant effect of time solely on rHb, with changes at both M1 (*p* = 0.0372) and M2 (*p* = 0.0018). Diabetes status itself, along with its interaction with time, did not significantly affect any of the O2C parameters. Independent t-tests comparing diabetic and non-diabetic patients identified a significant difference only in rHb at time point 4 at M2 (*p* = 0.0413). No other comparisons yielded significant results.

In a secondary analysis, we assessed the impact of insulin dependence (insulin-treated versus non-diabetic patients). Once again, time significantly influenced rHb (M1: *p* = 0.0436; M2: *p* = 0.0028), while insulin dependence and its interaction with time did not show significant effects. Corresponding t-tests verified that there were no significant differences between insulin-dependent diabetics and non-diabetics at any measured time point.

### 3.5. Changes in Microcirculation Among Patients with Critical Oxygen Saturation

We examined whether critical oxygen saturation (SO_2_ < 10%) at any of the five time points acted as a risk marker for impaired microcirculation. We compared long-term measurements of SO_2_, rHb, Flow, and Velocity between patients who experienced desaturation (*n* = 5) and those who did not (*n* = 25) using independent t-tests. Although no parameters showed statistically significant differences, Flow at M2 tended to be lower in patients with critical desaturation (*p* = 0.0706). Except for SO_2_ at M2, the mean values for all parameters were higher in patients without desaturation, though these differences did not reach significance.

We further evaluated shunt flow using ultrasound during long-term follow-up. The Wilcoxon two-sample test indicated a significant difference (*p* = 0.0449), with higher shunt flow in patients who experienced desaturation episodes. However, there was no significant difference in shunt flow on the first postoperative day. To identify clinical factors potentially associated with critical desaturation, we divided patients into two groups based on the presence or absence of SO_2_ < 10%. We found no significant differences in age, sex, height, weight, BMI, or in the presence of cardiovascular risk factors such as alcohol use, smoking, coronary artery disease, or hypertension. Interestingly, diabetes mellitus was significantly linked to the absence of critical desaturation (*p* = 0.0447), as none of the diabetic patients experienced critically low oxygen saturation.

### 3.6. Microcirculatory Changes in Patients Who Developed Ischemic Symptoms During Follow-Up

We compared microcirculatory parameters between patients who developed symptoms of hand ischemia during follow-up (*n* = 7) and those who remained asymptomatic (*n* = 10) using t-tests for SO_2_, rHb, Flow, and Velocity at both measurement sites (M1 and M2) across five time points (Time 1–5). Reported symptoms included exertional numbness (*n* = 1), exertional hand cramps (*n* = 1), resting numbness (*n* = 3), pallor and cold skin at rest (*n* = 1), and dialysis-associated hand pain (*n* = 1). Six patients met the criteria for DASS stage 1, and one patient met the criteria for DASS stage 2.

#### 3.6.1. SO_2_

**Figure 4:**
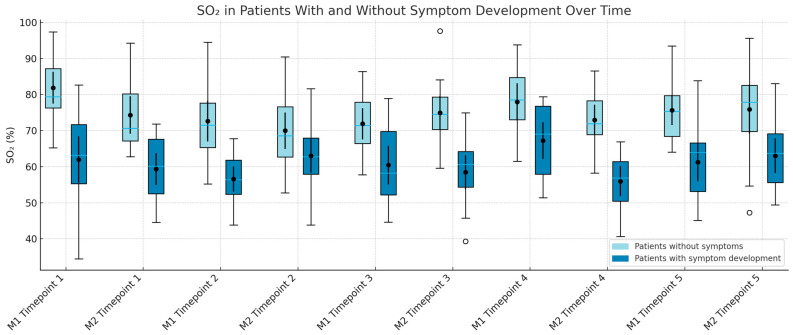
Boxplots showing regional oxygen saturation (SO_2_) over time in patients with and without symptom development. Measurements were taken at the thenar (M1) and hypothenar (M2) regions at five defined time points: preoperative (Timepoint 1), post-plexus block (Timepoint 2), intraoperative (Timepoint 3), first postoperative day (Timepoint 4), and long-term follow-up (Timepoint 5, 6–10 weeks). Light blue boxes represent patients without symptoms, and dark blue boxes represent patients with symptom development. Boxes indicate the interquartile range (IQR), horizontal lines indicate medians, whiskers show 1.5× IQR, and outliers are shown as individual points. Mean values and 95% confidence intervals are included for each group.

Asymptomatic patients consistently demonstrated higher SO_2_ values across all time points and sites, except at M1 during Time 3. Statistically significant differences were observed at Time 2 for both M1 (*p* = 0.0202) and M2 (*p* = 0.0157). SO_2_ values also became more variable after surgery, as shown in Figure 4.

#### 3.6.2. rHb

Although asymptomatic patients had higher rHb levels at Time 1–4 across both sites, none of the comparisons were statistically significant (*p* > 0.05).

#### 3.6.3. Flow

Symptomatic patients showed significantly lower Flow values immediately after surgery at M1 (*p* = 0.0402) and on postoperative day 1 at M2 (*p* = 0.0050). We also observed non-significant trends toward reduced Flow at M2 immediately post-op (*p* = 0.0825) and at M1 on POD 1 (*p* = 0.0748).

#### 3.6.4. Velocity

Velocity was significantly lower in symptomatic patients at M1 immediately after surgery (*p* = 0.0329) and at M2 on POD 1 (*p* = 0.0302). A trend toward lower values was also observed at M1 on POD 1 (*p* = 0.0820).

### 3.7. Predictive Analysis

**Figure 5:**
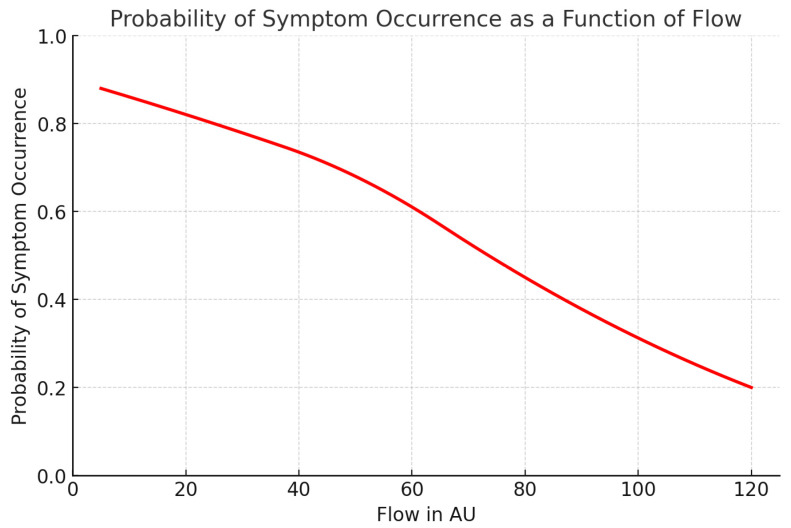
Probability of symptom occurrence as a function of flow. This graph illustrates the logistic regression analysis showing the probability of symptom development (e.g., Dialysis Access Steal Syndrome) as a function of measured microcirculatory flow (in arbitrary units, AU). A significant inverse relationship is observed: lower flow values are associated with a higher likelihood of symptom occurrence. The regression curve demonstrates that the probability of developing symptoms exceeds 80% when flow falls below approximately 30 AU, whereas values above 80 AU are associated with a symptom probability of less than 20%.

Given the strongest association for Flow at M2 on POD 1, we performed a logistic regression and ROC analysis to identify a predictive threshold. A cut-off value of 78.91 AU yielded 85.7% sensitivity and 80% specificity, suggesting strong predictive potential: 85.7% of patients who later developed symptoms could be identified early, with a 20% false-positive rate among asymptomatic individuals. Figure 5 below illustrates the probability of symptom development as a function of Flow.

### 3.8. Correlation Between O2C Parameters, Symptom Severity, and Shunt Flow

**Figure 6:**
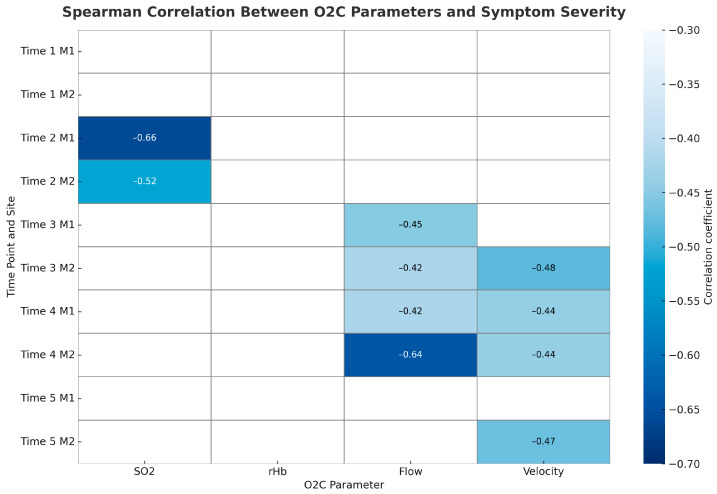
Spearman correlation between O2C and symptom severity. Darker shades of blue indicate stronger negative correlations.

To explore associations between long-term symptom development and microcirculatory measurements, we calculated Spearman correlation coefficients for each O2C parameter (SO_2_, rHb, Flow, Velocity) at both measurement sites (M1 and M2) across all five time points. We ranked symptom severity as follows: no symptoms, DASS stage 1, and DASS stage 2. We found that lower tissue oxygenation (SO_2_) measured after plexus anaesthesia (Time 2) significantly correlated with symptom severity at both sites, indicating that early preoperative desaturation may predict later ischemic symptoms. Flow and Velocity showed the strongest associations after surgery, especially at the hypothenar site (M2). On postoperative day 1 (Time 4), Flow at M2 significantly correlated with symptom severity, with similar trends observed for Velocity at M2 and Flow at M1. By long-term follow-up (Time 5), a trend toward reduced Velocity at M2 persisted in relation to symptom severity. Overall, patients with lower Flow and Velocity values, particularly at the hypothenar region, tended to have more severe symptoms, including DASS stages 1 and 2. Figure 6 displays the correlation heatmap illustrating the strength of Spearman correlations between O2C parameters and symptom severity across time points and measurement sites.

## 4. Discussion

### 4.1. Microcirculatory Effects of Plexus Anaesthesia

Our study confirms that axillary plexus anaesthesia has a measurable and consistent effect on the microcirculation of the upper limb. Following the regional blockade, we observed significant increases in SO_2_, flow, and velocity, while rHb levels mainly remained unchanged. These results support previous findings that the O2C system reliably detects improved tissue perfusion following nerve blocks [15] and are consistent with studies using similar optical technologies, which have also demonstrated reproducible changes in tissue perfusion and microcirculation after axillary plexus anesthesia across various modalities [16,17,18,19]. The increase in microcirculatory parameters likely results from vasodilation caused by sympathectomy and increased capillary recruitment, both of which enhance tissue oxygenation and perfusion [15,20]. These findings endorse the O2C device as a sensitive, non-invasive method for monitoring dynamic microvascular changes following regional anaesthesia [16,19,21]. The reproducibility of these effects at both thenar and hypothenar sites demonstrates the method’s reliability in a perioperative setting.

### 4.2. Hemodynamic Impact of Dialysis Access Surgery

Beyond the effects of plexus anaesthesia, creating a vascular access significantly altered local microcirculatory patterns. We observed postoperative increases in rHb and velocity, along with a trend toward higher flow. Interestingly, SO_2_, which initially increased after anaesthesia, decreased significantly following the surgical procedure. These results reflect complex hemodynamic shifts after fistula creation, likely involving both arterial steal and venous congestion phenomena. The isolated regional difference observed in rHb between M1 and M2 may indicate localized changes in capillary blood volume, further suggesting region-specific physiological responses. These findings emphasize the importance of spatial resolution in microcirculatory assessment. This aligns with current views on perioperative microcirculatory monitoring as described in recent reviews [19]. The ability of O2C to detect subtle, localized perfusion changes may provide an advantage over global hemodynamic indicators, particularly in assessing early or subclinical steal phenomena [15,20,22].

### 4.3. Microcirculatory Changes and Prognostic Implications for DASS

We observed that patients who developed ischemic symptoms during follow-up, most commonly DASS stage 1, showed lower Flow and Velocity values, especially at the hypothenar site (M2). The strongest predictive link was found for Flow at M2 on postoperative day 1, where a threshold of approximately 78.9 AU provided a sensitivity of 85.7% and a specificity of 80%. These findings indicate that early microcirculatory impairments could act as prognostic markers for DASS, particularly when assessed in anatomically distal regions. However, the relatively small sample size and significant interindividual variability limited the accuracy of predictions. Although Flow and Velocity trends are promising, the considerable overlap in values between symptomatic and asymptomatic patients emphasizes the need for multi-parametric or dynamic testing approaches. Future studies could improve discrimination by including contralateral measurements, fingertip probes, or provocation tests such as exercise or cold exposure.

### 4.4. Relationship Between Micro- and Macrovascular Assessments

We investigated whether microcirculatory values obtained with O2C correlate with macrocirculatory shunt flow measurements acquired via Doppler ultrasound. We observed a moderate correlation between flow at M1 on postoperative day 1 and ultrasound-measured shunt flow (r = 0.40), which could carry clinical relevance. In the past, micro- and macrocirculation were regarded as separate physiological systems. This dissociation has been documented in both dialysis and perioperative populations, where microcirculatory dysfunction can occur despite apparently normal systemic hemodynamics or conduit vessel flow [9,10,23,24]. Microcirculatory monitoring provides unique, tissue-level information about perfusion and oxygenation that is not captured by global or conduit vessel flow measurements. For example, microcirculatory impairment may precede or occur independently of changes in systemic parameters, and can be associated with adverse outcomes even when macrocirculatory targets are met [9,10,23,24]. This phenomenon, termed “loss of hemodynamic coherence,” is recognized in critical illness, perioperative care, and dialysis, and highlights the clinical value of direct microcirculatory assessment for early detection of regional ischemia and guiding individualized therapy [24,25]. We, however, think that with the aforementioned correlation, this should not be overstated as a strict dichotomy. The association suggests that microcirculatory measurements could provide complementary information to macroscopic flow assessment.

### 4.5. Discrepancies in Diabetic Patients

Contrary to expectations, diabetes mellitus did not significantly impair microcirculatory parameters in our cohort. We observed no consistent differences between diabetic and non-diabetic patients, and none of the diabetic patients exhibited critical SO_2_ desaturation (<10%) at any time point. This is a surprising and seemingly paradoxical finding, given the well-established link between diabetes and microvascular dysfunction in other organ systems. One possible explanation is the high degree of physiological heterogeneity within the diabetic group, including variations in disease duration, glycaemic control, and microvascular complications. Additionally, the small subgroup size and the presence of multiple comorbidities (e.g., hypertension, vascular disease) may have obscured subtle differences. The only statistically significant finding—higher rHb at M2 on postoperative day 1 in non-diabetics—was isolated and likely not clinically meaningful. However, it aligns with the literature, which highlights the need for larger, stratified studies to better understand the relationship between diabetes and cutaneous microcirculatory response in vascular access surgery [26,27,28,29]. Overall, these observations suggest that the relationship between diabetes and cutaneous microcirculation may be more complex than previously thought. While diabetic microangiopathy is well established in retinal and renal capillary beds, its effect on the forearm skin may vary depending on context and measurement conditions. Larger, stratified studies are necessary to determine whether diabetic patients genuinely differ in their microcirculatory response to vascular access creation.

### 4.6. Critical SO_2_ and Symptom Development

We also examined whether critical SO_2_ values (<10%) could predict clinical symptoms or indicate underlying risk. Patients who experienced such desaturation events showed no consistent demographic or clinical pattern, except for the absence of diabetes, which again questions traditional assumptions about diabetic microvascular risk. Although not statistically significant, this finding is consistent with our broader observation that diabetes did not increase the likelihood of impaired microcirculation in this group. Flow and velocity values remained lower at most time points in patients who later developed symptoms, supporting their potential as early signs of impending ischemia.

### 4.7. Case Observations

Two patients progressed to advanced DASS (stages 3 and 4) and required surgical revision. Interestingly, one of these patients showed elevated Flow preoperatively, while the other had increased SO_2_ postoperatively. These conflicting patterns, along with incomplete data, highlight a significant limitation of interpreting a single parameter. Even symptomatic patients may display “normal” or elevated microcirculatory values, emphasizing the need for a multimodal, context-sensitive approach when assessing DASS risk.

### 4.8. Limitations

This pilot study has several limitations. The small sample size (*n* = 31) limits statistical power for subgroup analyses (e.g., diabetes, ischemia, desaturation), increasing the risk of false-positive and false-negative findings. Although comparisons such as diabetes vs. non-diabetes revealed no significant differences in SO_2_ values, these results may be underpowered. Similarly, the logistic regression identifying flow as a potential predictor of DASS (AUC = 0.857) should be interpreted as exploratory, given the small number of symptomatic patients (*n* = 7).

Another key limitation is the lack of hematocrit and blood viscosity data. Both significantly affect microcirculatory oxygen delivery: hematocrit influences oxygen content via hemoglobin concentration, while increased viscosity may impair capillary flow. As the O2C device reflects both saturation and flow-dependent metrics, individual hematological profiles could confound interpretation. Future studies should include these parameters to enhance diagnostic validity.

Furthermore, long-term outcomes such as fistula maturation and patency were not systematically assessed, as the study focused on perioperative microcirculatory changes.

Lastly, no control group receiving general anaesthesia was included. Given that regional anaesthesia is standard at our center, and general anaesthesia poses ethical concerns in this population, the observational design reflects real-world practice but limits direct comparison of anaesthetic techniques.

All in all, our findings provide a basis for several future research directions. First, larger prospective studies with extended follow-up periods are needed to determine whether perioperative changes in microcirculatory parameters—such as SO_2_, Flow, and Velocity—are predictive of long-term AVF maturation, functional patency, or the development of complications such as Dialysis Access Steal Syndrome (DASS). Defining clinically meaningful thresholds or dynamic patterns in O2C measurements may allow for early identification of at-risk patients and guide intraoperative or postoperative management. Moreover, integrating microcirculatory monitoring with established macrocirculatory assessments, such as duplex sonography, could offer a more comprehensive understanding of vascular access physiology. Further studies should also explore the real-time application of O2C during surgery to inform intraoperative decision-making, as well as its comparative value across different anaesthetic techniques. Finally, multicenter trials with diverse patient populations and standardized measurement protocols will be essential to validate these findings and assess the feasibility of implementing microcirculatory monitoring as part of routine vascular access care.

## 5. Conclusions

This is the first study to assess perioperative microcirculatory dynamics using O2C in haemodialysis access surgery, demonstrating that Plexus anaesthesia reliably increases SO_2_, blood flow, and velocity. Additionally, lower flow measurements after surgery may predict the development of DASS. However, high individual variability limits clinical usefulness, and surprisingly, diabetes did not consistently negatively affect these microcirculatory parameters.

## Figures and Tables

**Table 1:** Patient demographics and anthropometric data.

Parameter	Mean	Minimum	Maximum
Age (years)	65.81	30	89
Body weight (kg)	77.87	45	120
Height (cm)	168.2	153	184
BMI (kg/m^2^)	27.69	18.3	46.9

BMI: Body Mass Index.

**Table 2:** Comorbidities and risk factors.

Comorbidity/Risk Factor	Prevalence *n* (%)
Diabetes Mellitus	15 (48.4%)
Insulin-Dependent Diabetes Mellitus History of Smoking	7 (22.6%) 15 (48.4%)
Peripheral arterial occlusive disease History of Alcohol abuse	4 (12.9%) 1 (3.2%)
Coronary artery disease	8 (25.8%)
Arterial hypertension ESRD	30 (96.8%) 16 (%)
Unknown cause of ESRD	16 (51.6%)
Known cause of ESRD -Polycystic kidney disease	15 (48.4%) 4 (12.9%)
-Partial nephrectomy	3 (9.7%)
-IgA nephritis	2 (−6.5%)
-Others (Undefined vasculopathy, Glomerulonephritis, history of nephritic syndrome, vancomycin toxicity)	6 (19.4%)

ESRD: End Stage Renal Disease.

**Table 3:** Procedural and postoperative characteristics.

Parameter	Value
Type of access creation	Primary: 23 patients Secondary: 8 patients
Type of vascular access	Gracz fistula: 19 Brachio-basilic fistula: 7 Brachiocephalic fistula: 2 Allogenous straight graft: 3
Side of access	Left arm: 19 Right arm: 12
Anesthesia	Regional: 30 General: 1
Mean procedure duration (minutes)	56.9 (Range: 35.0–100.0)
Mean fistula flow volume—POD1 (mL/min)	881.5 (Range: 350–1800; SD: 388.9)
Mean fistula flow volume—FU (mL/min)	1050.3 (Range: 500–2500; SD: 591.0)
Postoperative complications (first 8 months)	9 patients total -Type 2 stenosis: 7 -Shunt thrombosis: 1 -DASS: 1

DASS: Dialysis Associated Stea Syndrome; FU: Follow-up; POD1: Postoperative Day 1.

## Data Availability

The data can be obtained from the corresponding author upon reasonable request.
